# Ti nanoparticle additives enhance combustion behavior and reactivity in B-based thermites

**DOI:** 10.1039/d5ra02909k

**Published:** 2025-07-08

**Authors:** Yingke Chang, Wanjun Zhao, Enyi Chu, Jianxin Nie, Wei Ren, Buren Duan, Qingjie Jiao

**Affiliations:** a State Key Laboratory of Explosion Science and Safety Protection, Beijing Institute of Technology Beijing 100081 China wanjunzhaowj@bit.edu.cn; b State Key Laboratory of Transient Chemical Effects and Control, Shaanxi Applied Physics and Chemistry Research Institute Xi'an 710016 China; c Liaoning North Huafeng Special Chemical Limited Company Fushun 113003 China

## Abstract

Owing to its high energy density, boron attracts much attention as a fuel in energetic materials. However, its reactivity is limited by the liquid oxide shell during combustion. In this study, we have incorporated Ti nanoparticles into B-fueled thermites with high-oxygen-content potassium oxysalts as the oxidizers. The combustion behavior was characterized *via* pressure cell and a calorimeter. By evaluating the thermal behavior of thermites, the ignition mechanism of B-fueled and Ti-fueled thermites was revealed. Compared to single-component thermites, the B/Ti binary thermites demonstrated peak pressures that were approximately 1.5 to 3 times higher and exhibited significantly enhanced reactivity. Additionally, they showed an 8–18% increase in combustion efficiency over pure boron-based thermites. Thermal characterization results showed that the onset reaction temperature of B/Ti-based ternary thermites could be effectively tuned by varying the Ti content. Therefore, Ti nanoparticles are effective additives for B-based energetics for enhancing combustion behavior and tuning the reaction temperature.

## Abstract 2

The reaction mechanism of boron-based thermites can be divided into the condensed and gas phase mechanism.

## Graphical abstract

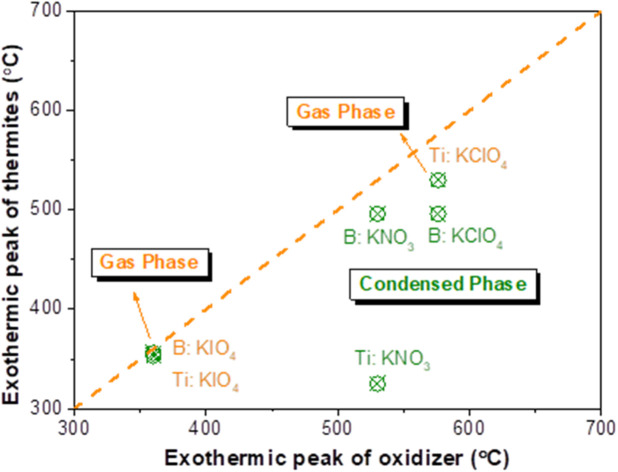

## 1. Introduction

Aluminum (Al), boron (B), silicon (Si), and titanium (Ti) nanoparticles have always attracted much research attention for their application in energetic materials, including pyrotechnics, propellants, and explosives.^1–5^ Boron has remained an appealing fuel because of its high energy density and excellent energetic behavior.^6–8^ However, the low melting point of boron oxide (B_2_O_3_) leads to an impervious liquid shell, which prohibits efficient combustion of boron.^9,10^ In addition, the formation of HOBO in hydrogen-containing atmosphere restrains the combustion of boron.^11,12^ It is well documented that the combustion of boron can be divided into two stages, which are the slow oxidization of the boron core in the presence of B_2_O_3_ and fast burning after the removal of the oxide shell.^13–15^ Thus, a large body of work has been conducted to either promote the flame temperature for evaporating the B_2_O_3_ shell by adding the widely used fuel Al as the binary fuel^16^ or introducing metal oxides to catalyze the oxidation of B.^17–19^ Meanwhile, the incorporation of other metals such as magnesium (Mg) and iron (Fe) remains an effective way to promote the energetic behavior of B.^20,21^ The incorporation of Mg into B could effectively increase the oxidation rate and decrease the onset reaction temperature by alleviating the prohibiting effect of the oxide layer.^20^ Cheng *et al.*^21^ electrosprayed nitrocellulose (NC) and iron (Fe) particles to obtain B/NC/Fe particles and achieved a lower oxidation temperature than pure boron. Trunov *et al.* achieved efficient combustion of B–Ti, which outperforms Al in wet gaseous and dry environments^22^ because of the exothermic boron–titanium reaction. Ti nanoparticles have attracted attention recently owing to its easy ignition in air. They have been used as a fuel in energetic materials and as additives in thermites to effectively tune the ignition temperature and energetic behavior.^23–28^

Potassium oxysalts have been widely used in thermites owing to their low bond energy of nonmetal–oxygen pair and high oxygen content.^29^ Jian *et al.*^30^ applied potassium periodate (KIO_4_) as the oxidizer in nano-energetic gas generators. Zhou *et al.*^31^ systematically investigated the reaction mechanism of thermites involving Al and several potassium oxysalts. Results demonstrate that the gas-phase mechanism dominates for the reactive thermites, whereas the reaction mechanism of less reactive thermites is a condensed phase mechanism. However, the reaction mechanism of B- and Ti-based thermites with potassium oxysalts has not been widely reported and discussed.^32^ Furthermore, the incorporation of Ti as a combustion modifier for boron B-based systems has received limited scientific attention. Thus, the underlying synergistic mechanisms driving their thermochemical interactions remain poorly characterized at both macro- and micro-scales.

In this work, we have chosen well-performing potassium oxysalts with low oxygen release temperature as the oxidizers for B-based thermites, considering the low oxygen release temperature of KIO_4_, and wide application of potassium perchlorate (KClO_4_) and potassium nitrate (KNO_3_).^33,34^ To improve their combustion behavior, Ti nanoparticles were incorporated, and the energetic performance was characterized *via* combustion tests and calorimetry. The thermal properties of thermites have been investigated to measure the onset reaction temperature; thus, the reaction mechanism of B-based, Ti-based, and binary fuels-based thermites with potassium oxysalts has been explored.

## 2. Experimental section

### 2.1 Caution

Standard precautions, including face shield, earplugs, and leather gloves, should be taken during the preparation, handling, and measurement of thermites. Especially when using Ti nanoparticles, the operator should be extremely careful since the active Ti nanoparticles are sensitive to static electricity.

### 2.2 Preparation of thermites

Boron nanopowders (∼60 nm) and nano titanium particles (∼50–80 nm), both with 70 wt% active content, were purchased from the BGRIMM Advanced Materials Science & Technology Corporation and Sigma-Aldrich Corporation, respectively. Thermogravimetry analysis (TGA) was applied to determine the active metal contents of the above powders. The oxidizers used in this study include KClO_4_, KNO_3_, and KIO_4_ available from Sinopharm Chemical Reagent Corporation. The stoichiometric ratio and expected energy release of fuels and oxidizers were determined based on eqn (1)–(6):

1 8B + 3KClO_4_ → 4B_2_O_3_ + 3KCl, Δ*H* = −9231 J g^−1^

2 2Ti + KClO_4_ → 2TiO_2_ + KCl, Δ*H* = −8089 J g^−1^

3 8B + 3KIO_4_ → 4B_2_O_3_ + 3KI, Δ*H* = −5445 J g^−1^

4 2Ti + KIO_4_ → 2TiO_2_ + KI, Δ*H* = −5031 J g^−1^

5 4B + 2KNO_3_ → 2B_2_O_3_ + 2K + N_2_, Δ*H* = −6333 J g^−1^

6 3Ti + 2KNO_3_ → 3TiO_2_ + 2K + N_2_, Δ*H* = −5327 J g^−1^

The formulations for the thermites are shown in Table 1. The molar percentage of B in the binary fuel is 30%, 50%, and 70%. 70B/30Ti:KClO_4_ in Table 1 indicates a 1 : 1 molar ratio of fuel to oxidizer, with the fuel consisting of 70% B and 30% Ti by molar percentage, respectively. The KClO_4_ content is determined by a chemical equivalence ratio of 1.0.

**Table 1:** Formulations of B/Ti:KClO_4_ thermites

Thermites	B (wt%)	Ti (wt%)	KClO_4_ (wt%)
B:KClO_4_	17.5	0.0	82.5
70B/30Ti:KClO_4_	7.7	14.5	77.8
50B/50Ti:KClO_4_	6.3	27.6	66.1
30B/70Ti:KClO_4_	3.4	34.8	61.8
Ti:KClO_4_	0.0	43.2	56.8

The weighed fuels and oxidizers were put into a vial with ∼10 mL hexane and sonicated for 30 min, which was followed by drying overnight in a hood. The dry powders were gently broken apart by a spatula to obtain loose powders for further measurement. The morphology of the thermites was characterized by scanning electron microscopy and energy dispersive spectroscopy (SEM/EDS, Hitachi S-4800, Japan).

### 2.3 Measurement of reactivity

The reactivity of the thermites was characterized *via* the combustion cell test.^35^ Normally, ∼50 mg samples were loaded in a combustion cell of ∼50 cm^3^ volume. Then, the samples were ignited *via* the nichrome above them. The pressure peaks, pressurization rate, and burn time could then be obtained based on the pressure change with time. In detail, the pressurization rate is determined by calculating the slope of the initial pressure rise. The burn time is evaluated based on the width at half-max of the optical emission. The combustion test for each sample is repeated in triplicate, and the average values with error bars that represent the standard deviation are presented.

### 2.4 Combustion heat release measurement

The heat release during the reaction of thermites was measured *via* calorimetry. Typically, ∼200 mg of thermites were put into a sealed calorimeter and then ignited by the nichrome wire above them. The heat released from the thermites heated the water in the calorimeter. The combustion heat of the samples could be then calculated and obtained.

### 2.5 Flame temperature measurement

Typically, ∼30 mg composites were weighed out and placed on a stage, which was then ignited by a CO_2_ laser ignition system with an output power of 100 W and a duration of 1000 ms. The flame temperature of the thermistor is evaluated by means of an infrared thermometer (SC300, Sweden), which determines the temperature of an object by measuring the infrared radiation it emits.

### 2.6 Thermal behavior characterization

The onset reaction temperature of the thermites was evaluated by differential scanning calorimetry (DSC, Netzsch STA 449 F3) test. Usually, ∼2 mg thermites were loaded into the closed crucible and heated at a heating rate of 20 °C min^−1^ with 100 L min^−1^ Ar flow from room temperature to 600 °C. Three replicates of each formulation were analyzed.

## 3. Results and discussion

### 3.1 Thermochemical calculation

**Fig. 1:**
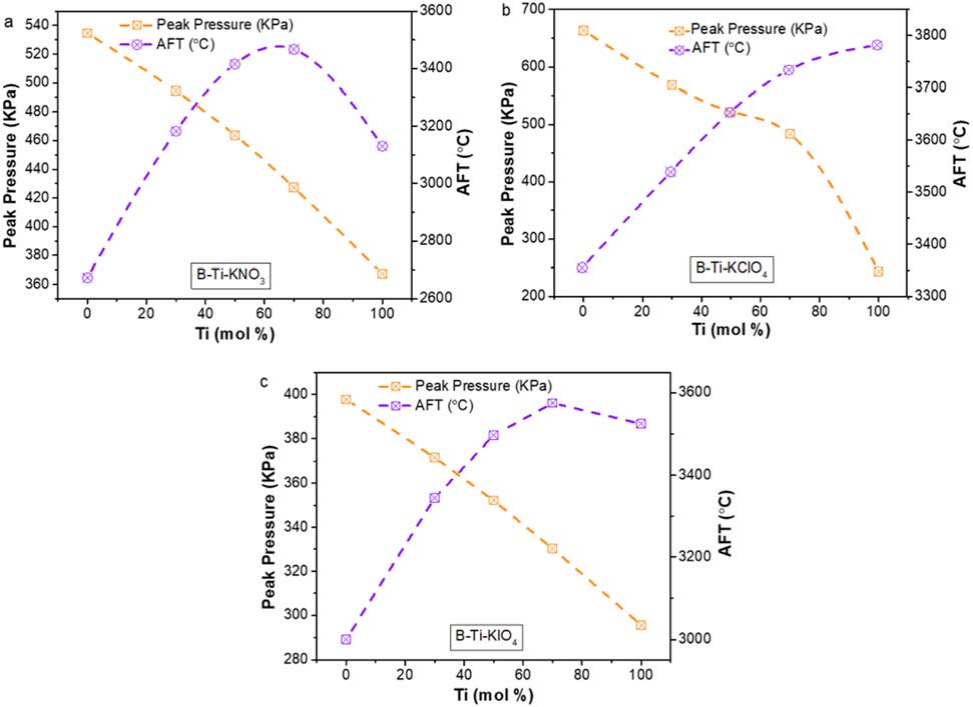
Theoretical peak pressure and AFT of (a) B/Ti:KNO_3_, (b) B/Ti:KClO_4_, and (c) B/Ti:KIO_4_ thermites.

The combustion calculations were conducted based on the free energy minimization technique to predict the influence of Ti addition on the peak pressure and the adiabatic flame temperature (AFT) of the B-fueled thermites.^36^ As demonstrated in Fig. 1, the incorporation of Ti can raise the AFT of the B-based thermites. For example, upon adding 70% Ti to pure B/KNO_3_, the AFT of the thermite was increased from 367 °C to 528 °C. Thus, it is reasonable to speculate that the incorporation of Ti could raise the flame temperature of B-based thermites. This is helpful for the boiling of B_2_O_3_, and thus results in enhanced reactivity. Conversely, the addition of Ti monotonously reduces the peak pressure. This is caused by the fact that the gas production per mass of Ti-based thermites is less than that for B-based ones.

### 3.2 Morphology characterization results

**Fig. 2:**
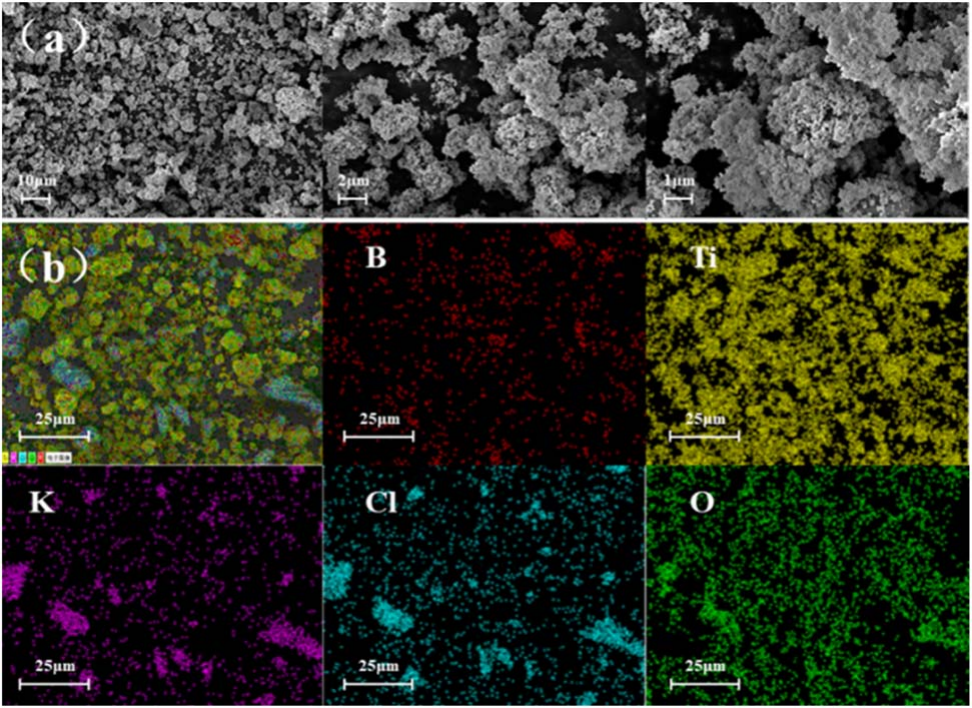
(a) SEM images and (b) EDS results of B/Ti:KClO_4_.

The morphology of the composites was evaluated using SEM. The SEM images of the B/Ti binary thermites, as shown in Fig. 2a, indicate that the small particle sizes of the nanosized B and Ti contributed to their adherence to KClO_4_. In the B/Ti binary thermites, B, Ti, and KClO_4_ were mixed uniformly with one another. The EDS results presented in Fig. 2b indicate that B, Ti, and K, C, O are well distributed in the B/Ti/KClO_4_ thermites.

### 3.3 Combustion test results of B/Ti-fueled thermites

**Fig. 3:**
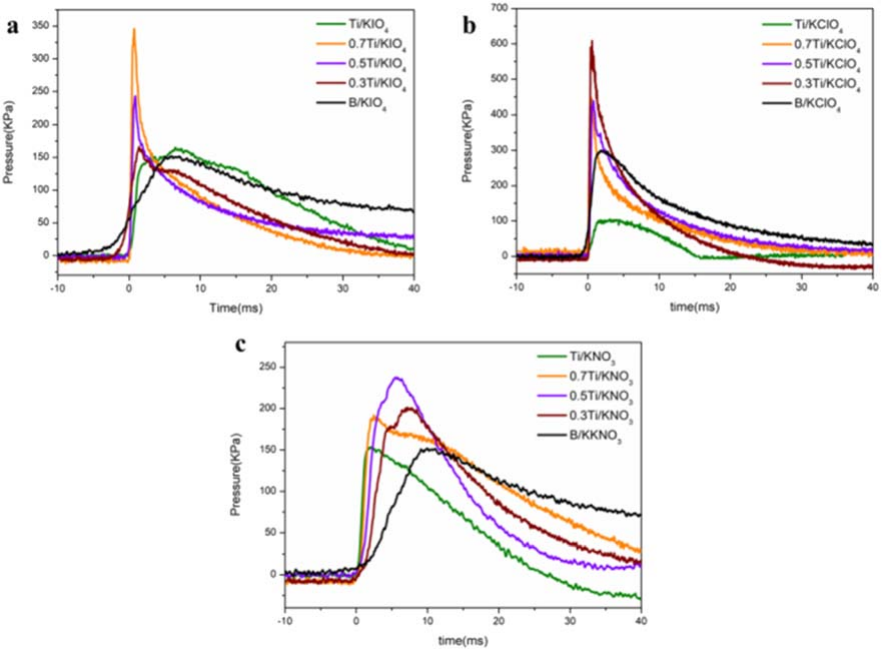
Combustion test results of (a) B/Ti:KIO_4_, (b) B/Ti:KClO_4_, and (c) B/Ti:KNO_3_.

The combustion test results (Fig. 3) demonstrate that the energetic performance of the B/Ti-binary fueled thermites is superior to that of the pure B-based and pure Ti-based thermites. In detail, Fig. 3a reveals that the peak pressure of 50B/50Ti:KNO_3_ is ∼1.5 times that of B:KNO_3_ and Ti:KNO_3_. For the B/Ti:KClO_4_ ternary system, the incorporation of 30 mol% Ti can raise the peak pressure of B:KClO_4_ by a factor of 2, which changes from ∼300 kPa to ∼700 kPa. The highest peak pressure (360 ± 60 kPa) of B/Ti:KIO_4_ is achieved by 30B/70Ti:KIO_4_, which is ∼3 times greater than that for the B:KIO_4_ and Ti:KIO_4_ binary systems. This is not consistent with the calculated trend shown in Fig. 1, indicating that the addition of Ti plays a positive role in the combustion of B, which will be discussed later.

**Fig. 4:**
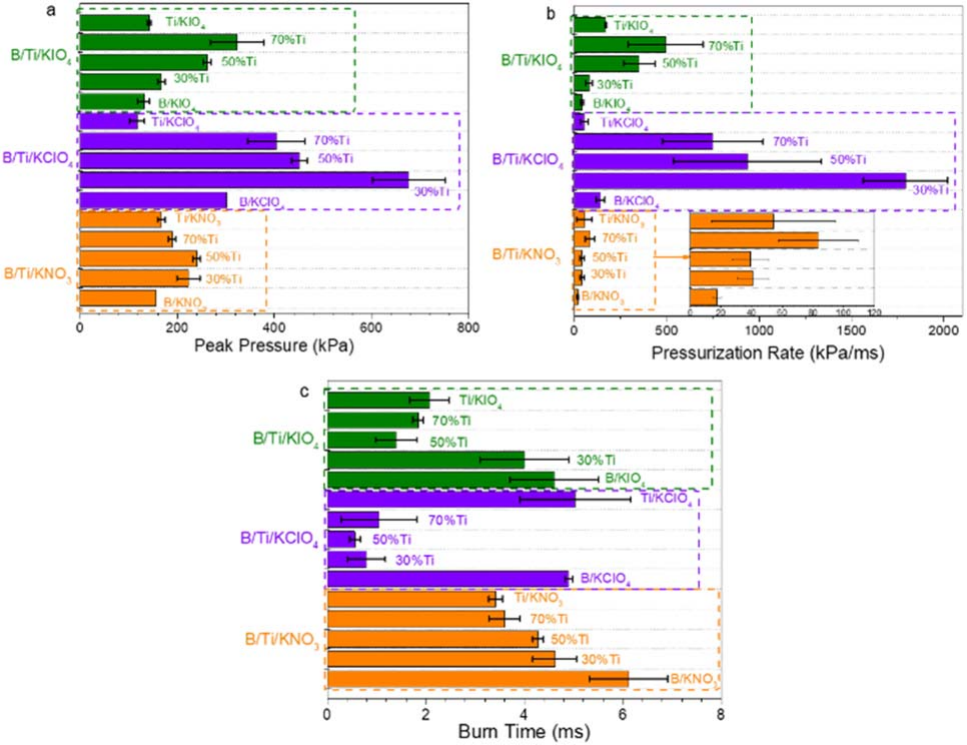
(a) Peak pressure, (b) pressurization rate, and (c) burn time of B/Ti:potassium oxysalt.

The pressurization rate, which usually represents the reactivity of thermites,^37^ of the B/Ti:potassium oxysalts systems is significantly higher than that of B/oxidizers and Ti/oxidizers (Fig. 4b). The reactivity of B:KNO_3_ has been raised from ∼20 kPa ms^−1^ to ∼80 kPa ms^−1^*via* incorporation of 70% Ti. The pressurization rate of the 30B/70Ti:KIO_4_ (∼1800 kPa ms^−1^) system is more than one order of magnitude larger than that of B:KIO_4_. Similarly, the reactivity of the 70B/30Ti:KClO_4_ system is ∼12 times and 35 times higher than that of the B:KClO_4_ and Ti:KClO_4_ systems, respectively. Correspondingly, a significantly shorter burning time of the B/Ti-fueled systems compared to monometal-based systems has been observed, which is a general corresponding relationship between a shorter burn time and higher peak pressure or reactivity (Fig. 4c).

Among different potassium oxysalts-based systems, the best combustion performance was achieved by the thermites with KClO_4_ as the oxidizer. The higher reactivity and peak pressure can be contributed to the fast decomposition rate of KClO_4_ and higher reaction enthalpy of KClO_4_ compared to that for KNO_3_ and KIO_4_, respectively.

### 3.4 Flame temperature evaluation

**Fig. 5:**
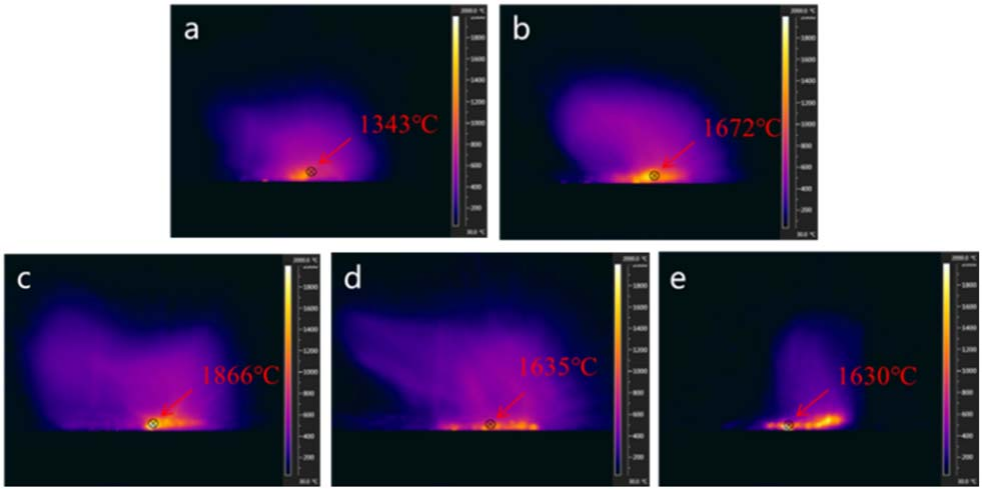
Infrared radiation images of the burning process of (a) B:KNO_3_, (b) 70B/30Ti:KNO_3_, (c) 50B/50Ti:KNO_3_, (d) 30B/70Ti:KNO_3_, and (e) Ti:KNO_3_.

Since the theoretical calculation in Fig. 1 indicates the flame temperature provided by the reaction of Ti and oxidizers is raised as predicted, we measured the flame temperature of B/Ti binary fueled thermites by taking B/Ti:KNO_3_ as an example. The infrared radiation images in Fig. 5 demonstrate that the flame temperature of ternary thermites is higher than that for the B- and Ti-based binary energetic composites. In detail, the flame temperature of the B:KNO_3_ composites is raised from ∼1343 °C to ∼1672 °C with the incorporation of 30 mol% Ti. The highest flame temperature of ∼1866 °C was achieved by the 50B/50Ti:KNO_3_ thermites. As for 70B/30Ti:KNO_3_, the flame temperature is close to that of Ti:KNO_3_. Thus, it can be concluded from infrared radiation results that the incorporation of Ti can effectively raise the flame temperature of B-based thermites.

Thus, the greatly enhanced peak pressure and reactivity of B-fueled thermites achieved *via* the addition of Ti particles could be attributed to the combination of the increased flame temperature resulting from the reaction between Ti and oxidizers and the pathway created by the TiO_2_/B_2_O_3_ mixture. Namely, the higher flame temperature results in faster evaporation of the B_2_O_3_ shell, which promotes the reaction between B and oxidizers. In the meanwhile, with the addition of Ti particles, the potassium oxysalts react with the B core with lower resistance compared to the resistance created by the liquid oxide shell. Therefore, with the addition of Ti nanoparticles, the potassium oxysalts can react more easily and rapidly with B, resulting in a dramatically higher peak pressure and reactivity. According to eqn (1)–(6), the reaction enthalpy between B and the potassium oxysalts is higher than that of Ti and the corresponding oxidizers. Thus, both peak pressure and the pressurization rate of the B/Ti binary fuels-based thermites are higher than those of the B- and Ti-based energetics.

### 3.5 Heat release and combustion efficiency

**Fig. 6:**
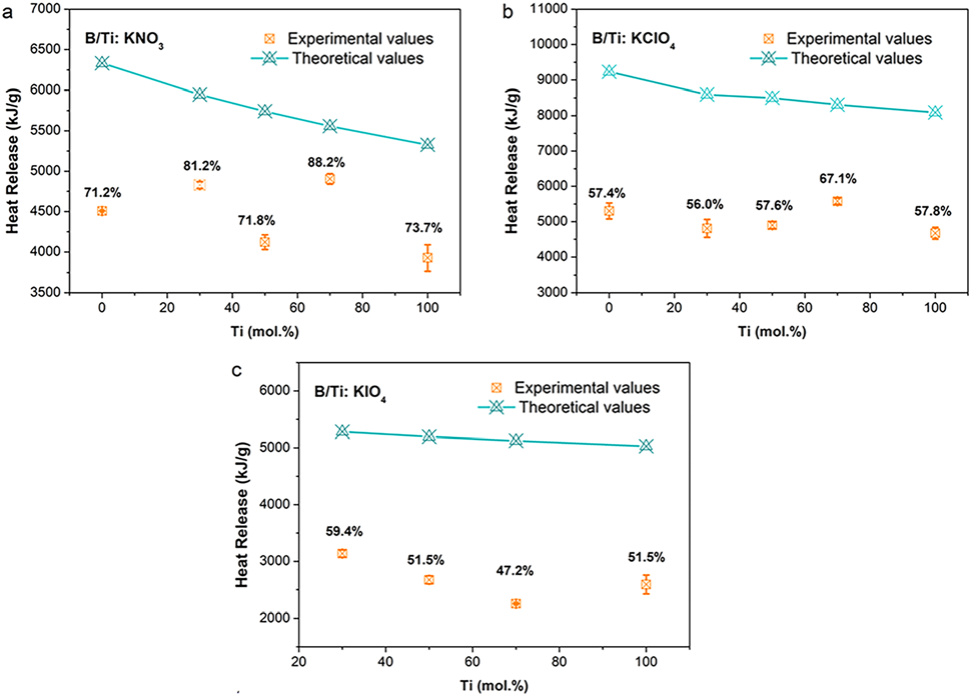
Experimental and theoretical heat release of (a) B/Ti:KNO_3_, (b) B/Ti:KClO_4_, and (c) B/Ti:KIO_4_. Note: B:KIO_4_ cannot be ignited in the calorimeter.

As demonstrated in Fig. 6, the combustion heat release by the reaction of ternary thermites is higher than that for pure Ti-based binary thermites, which is reasonable since the theoretical heat release of B-based thermites is higher than that for Ti-fueled ones according to eqn (1)–(6). Different from the trend of the theoretical heat release, the highest heat release was achieved by the B/Ti-based ternary thermites, including 30B/70Ti:KNO_3_ (∼4900 kJ g^−1^), 30B/70Ti:KClO_4_ (∼5580 kJ g^−1^), and 70B/30Ti:KIO_4_ (∼3140 kJ g^−1^), which is ∼400 kJ g^−1^, ∼280 kJ g^−1^, and ∼540 kJ g^−1^ higher than that of corresponding pure boron or pure Ti-based energetics, respectively. The combustion efficiency has also been promoted by ∼8–18% when compared to pure B or Ti-based thermites. The combustion efficiency is defined as the ratio of the experimentally measured heat release and the theoretical value. The highest heat release of ternary thermites may be attributed to the incorporation of Ti promoting the combustion of B.

### 3.6 Thermal behavior and reaction mechanism

**Fig. 7:**
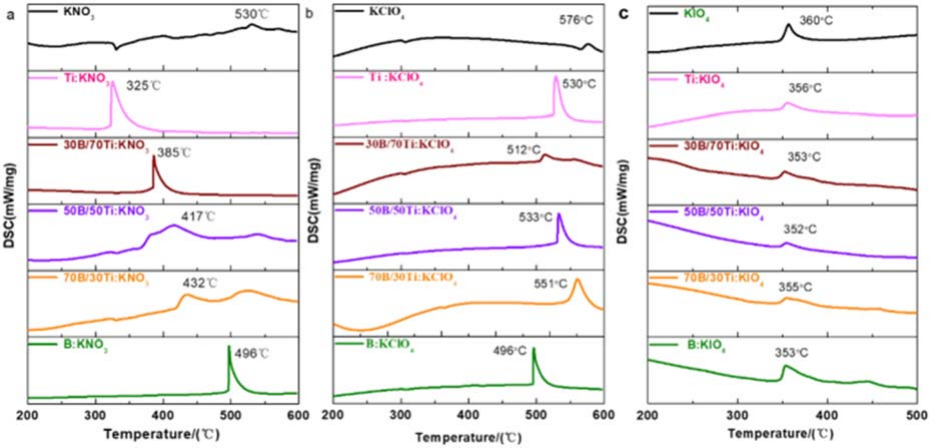
DSC curves of (a) B/Ti:KNO_3_, (b) B/Ti:KClO_4_, and (c) B/Ti:KIO_4_ thermites.

The reaction between fuels and oxidizers produces heat, which is reflected in the exothermic peaks in DSC curves. The exothermic peak temperature reflects the reaction temperature of the thermites. As shown in Fig. 7a, the exothermic peak temperature decreases monotonically from 496 °C to approximately 325 °C with increasing Ti content in the B/Ti:KNO_3_ ternary system. This indicates that the incorporation of Ti into B-based thermites enhances the reaction between the fuel and KNO_3_. This may be attributed to the fact that the reaction between Ti and KNO_3_ releases heat and initiates the reaction of B and KNO_3_. As for B/Ti:KIO_4_, the exothermic peak temperature is ∼355 °C, which is nearly the same as the exothermic peak caused by the decomposition of KIO_4_. The similar exothermic peak temperature for B/Ti:KIO_4_ indicates that once KIO_4_ decomposes, the reaction of the thermites can be initiated, which reveals the condensed phase mechanism for thermites with KIO_4_ as the oxidizer.

Based on the relationship between the oxygen release temperature and the reaction temperature of the corresponding thermites, we have concluded the reaction mechanism of B-based and Ti-based thermites. Thus, we infer that the reaction mechanism of boron-based thermites may be divided into the condensed phase and the gas phase mechanism. When the oxygen release temperature of the oxidizer is lower than the melting point of B_2_O_3_ (∼450 °C), the oxidization of the boron can occur once the oxygen is available to react with the boron core without the inhibition of the impervious B_2_O_3_ shell. Thus, the reaction mechanism of B:KNO_3_ and B:KClO_4_ is a condensed phase since the exothermic temperature of KNO_3_ (∼530 °C) and KClO_4_ (∼576 °C) is higher than that of B:KNO_3_ and B:KClO_4_ (both at ∼496 °C). As for B:KIO_4_, the exothermic peak for the reaction (∼353 °C) is nearly the same as that for KIO_4_ (∼360 °C), which indicates that the reaction between B and KIO_4_ is initiated once KIO_4_ decomposes and provides oxygen to react with B.

**Fig. 8:**
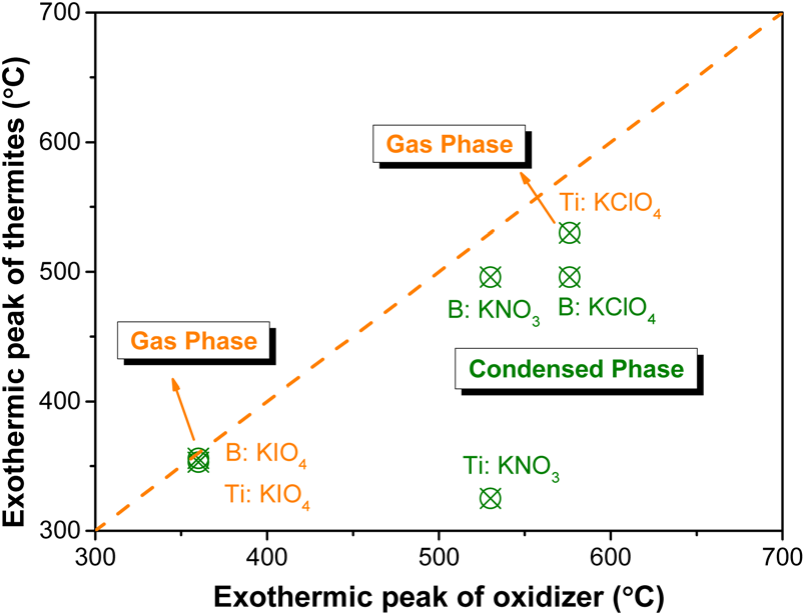
Ignition mechanism of B and Ti-based thermites.

As for the Ti-based thermites, the condensed phase mechanism dominates for Ti:KNO_3_ since the exothermic peak of Ti:KNO_3_ is ∼200 °C lower than that of KNO_3_ (∼325 °C). For Ti:KIO_4_, the exothermic peak for the reaction is close to that of KIO_4_, indicating a gas phase mechanism. The reaction of Ti:KClO_4_ proved to be dominated *via* the gas phase mechanism^5^ even though the exothermic peak of KClO_4_ is ∼50 °C lower than that of Ti:KClO_4_. Therefore, we summarize the ignition mechanism of the B and Ti-based thermites, as shown in Fig. 8.

## 4. Conclusion

In this paper, Ti nanoparticles were incorporated into B-fueled thermites with potassium oxysalts as the oxidizers. Pressure cell test results demonstrate that the peak pressure and reactivity of the B/Ti-based thermites are higher than that for the pure B and Ti-based energetics. Correspondingly, the highest heat release and combustion efficiency are achieved by ternary systems instead of binary systems. The enhanced combustion performance *via* the incorporation of Ti can be attributed to the increased flame temperature by the reaction of Ti and oxidizers and the low-resistance pathway for oxygen created by the B_2_O_3_/TiO_2_ mixture. Thermal tests were conducted to investigate the reaction mechanism of the B- and Ti-based thermites. The results show that the reaction temperature of B:KNO_3_ and B:KClO_4_ could be tailored effectively by the addition of Ti. Based on the thermal test results, we also concluded that the reaction mechanism of B:KNO_3_, B:KClO_4_, and Ti:KNO_3_ is a condensed phase. Therefore, Ti particles are a promising candidate for tuning and/or enhancing the reaction and combustion between B and potassium oxysalts.

## Author contributions

Yingke Chang: conceptualization and writing–original draft. Wanjun Zhao: methodology, writing–review and editing. Enyi Chu: investigation. Jianxin Nie: investigation. Wei Ren: data curation. Buren Duan: methodology. Qingjie Jiao: project administration.

## Conflicts of interest

The authors declare that they have no known competing financial interests or personal relationships that could have appeared to influence the work reported in this paper.

## Data Availability

The data supporting the findings of this study are available upon reasonable request from the corresponding author.
