# Correction: Ti nanoparticle additives enhance combustion behavior and reactivity in B-based thermites

**DOI:** 10.1039/d5ra90107c

**Published:** 2025-09-26

**Authors:** Yingke Chang, Wanjun Zhao, Enyi Chu, Jianxin Nie, Wei Ren, Buren Duan, Qingjie Jiao

**Affiliations:** a State Key Laboratory of Explosion Science and Safety Protection, Beijing Institute of Technology Beijing 100081 China wanjunzhaowj@bit.edu.cn dbr@njust.edu.cn; b State Key Laboratory of Transient Chemical Effects and Control, Shaanxi Applied Physics and Chemistry Research Institute Xi’an 710016 China; c Liaoning North Huafeng Special Chemical Limited Company Fushun 113003 China

## Abstract

Correction for ‘Ti nanoparticle additives enhance combustion behavior and reactivity in B-based thermites’ by Yingke Chang *et al.*, *RSC Adv.*, 2025, **15**, 23867–23873, https://doi.org/10.1039/D5RA02909K.

The authors regret that Buren Duan was not indicated as a corresponding author in the original article. The corrected author list is as shown herein.

The Royal Society of Chemistry apologises for these errors and any consequent inconvenience to authors and readers.

